# *Toxoplasma* GRA15 Activates the NF-κB Pathway through Interactions with TNF Receptor-Associated Factors

**DOI:** 10.1128/mBio.00808-19

**Published:** 2019-07-16

**Authors:** Lamba Omar Sangaré, Ninghan Yang, Eleni K. Konstantinou, Diana Lu, Debanjan Mukhopadhyay, Lucy H. Young, Jeroen P. J. Saeij

**Affiliations:** a School of Veterinary Medicine, Department of Pathology, Microbiology and Immunology, University of California Davis, Davis, California, USA; b Department of Ophthalmology, Harvard Medical School, Boston, Massachusetts, USA; c Department of Biology, Massachusetts Institute of Technology, Cambridge, Massachusetts, USA; Albert Einstein College of Medicine

**Keywords:** NF-κB pathway, TRAF2, TRAF6, *Toxoplasma gondii*, host-pathogen interactions

## Abstract

The protozoan parasite Toxoplasma gondii secretes proteins from specialized organelles, the rhoptries, and dense granules, which are involved in the modulation of host cell processes. Dense granule protein GRA15 activates the nuclear factor kappa B (NF-κB) pathway, which plays an important role in cell death, innate immunity, and inflammation. Exactly how GRA15 activates the NF-κB pathway is unknown. Here we show that GRA15 interacts with tumor necrosis factor receptor-associated factors (TRAFs), which are adaptor proteins functioning upstream of the NF-κB transcription factor. We identified several TRAF binding sites in the GRA15 amino acid sequence and showed that these are involved in NF-κB activation. Furthermore, a TRAF2 knockout cell line has impaired GRA15-mediated NF-κB activation. Thus, we determined the mechanism for GRA15-dependent NF-κB activation.

## Abstract (precis)

The parasite *Toxoplasma* can cause birth defects and severe disease in immunosuppressed patients. Strain differences in pathogenicity exist, and these differences are due to polymorphic effector proteins that *Toxoplasma* secretes into the host cell to coopt host cell functions. The effector protein GRA15 of some *Toxoplasma* strains activates the nuclear factor kappa B (NF-κB) pathway, which plays an important role in cell death, innate immunity, and inflammation. We show that GRA15 interacts with TNF receptor-associated factors (TRAFs), which are adaptor proteins functioning upstream of the NF-κB transcription factor. Deletion of TRAF-binding sites in GRA15 greatly reduces its ability to activate the NF-κB pathway, and TRAF2 knockout cells have impaired GRA15-mediated NF-κB activation. Thus, we determined the mechanism for GRA15-dependent NF-κB activation.

## INTRODUCTION

*Toxoplasma* is an obligate intracellular protozoan parasite that can cause congenital toxoplasmosis, opportunistic infections in immunosuppressed patients, and ocular disease (1). There are many different *Toxoplasma* strains, and dramatic strain differences in virulence and modulation of the host cell exist (2). In Europe and North America, most human infections are caused by type II strains, but type III, type 12, and type I strains are also present in these regions (3, 4). To modulate its host cells, *Toxoplasma* secretes specialized effector proteins called ROPs and GRAs from organelles known as the rhoptries and dense granules, respectively (2). Some GRAs are secreted beyond the parasitophorous vacuole (PV) membrane (PVM) into the host cell cytosol, most likely through a PVM-localized translocon consisting of the *Toxoplasma* proteins MYR1/2/3 (5, 6), and modulate host cell signaling pathways (7–10). Other GRAs are inserted in the parasitophorous vacuole membrane (PVM), with one part facing the host cytoplasm to interact with host proteins, often in a strain-specific manner. For example, GRA6 from type I and III strains (GRA6_I/III_) activates the NFAT4 (nuclear factor of activated T cells) transcription factor (11), MAF1bI/III (mitochondrial association factor 1b_I/III_) mediates host mitochondrial association with the PVM, GRA7 binds to IRGa6 (GTP-bound immunity-related GTPase a6) (12, 13), and GRA15_II_ activates the NF-κB pathway (14).

The NF-κB pathway consists of the canonical and alternative pathways, with the former involved in innate, inflammatory, and adaptive immune responses, while the latter is involved in the regulation of lymphoid organ development, B cell function, and adaptive immunity. Five NF-κB genes can produce five transcription factors (RelA, cRel, RelB, p50, p52), which can form 15 transcription factors through homo- and heterodimerization [e.g., RelA (p65/p50)] (15). Given the crucial role NF-κB plays in cell death, inflammation, and the immune response, many pathogen effector proteins target the NF-κB pathway (16). *Toxoplasma* GRA15 accounts for differences in NF-κB activation between different isolates of the type I clonal lineage (RH and GT1) (17) and between type II and type III strains upon infection (14). GRA15 can stimulate the host innate immune response through mediating human monocyte secretion of the NF-κB-dependent cytokine interleukin-1β (IL-1β) (18), the induction of IL-12 secretion by murine macrophages (14, 19), and upregulation of the CD40 cell surface receptor on murine macrophages, which leads to IL-12 production and induction of the interferon gamma (IFN-γ) response (20). GRA15-mediated inflammation can also lead to miscarriage and stillbirth in pregnant mice (21). The cytokine IFN-γ upregulates the large family of GTPases named the guanylate binding proteins (GBPs), which can bind to the PVM and mediate its destruction and subsequently that of the parasite inside (22). GRA15 enhances the recruitment of murine GBP1 to the PVM through an unknown mechanism (23, 24). In a human monocyte-hepatocyte coculture system, it was recently shown that GRA15-induced IL-1β secretion by monocytes leads to inducible nitric oxide synthase (iNOS) upregulation in hepatocytes. The nitric oxide (NO) produced inhibits the IFN-γ-induced enzyme indoleamine-2,3-deoxygenase (IDO), which degrades l-tryptophan for which *Toxoplasma* is auxotrophic, resulting in enhanced parasite growth (25). Thus, GRA15 has pleiotropic effects on host immune responses to *Toxoplasma*.

Strain differences in the activation of NF-κB could be due to GRA15 amino acid differences (14), differences in GRA15 expression levels (19), other effectors that modulate the NF-κB pathway (19), or a combination of all of these. GRA15-dependent NF-κB activation was Myd88/TRIF (adaptors directly downstream of Toll-like receptors [TLRs]) independent but partially TRAF6 (tumor necrosis factor [TNF] receptor-associated factor 6) dependent (14). However, the exact mechanism of GRA15-dependent NF-κB activation remains unclear. Here we show that both type II and type III GRA15 proteins are sufficient to cause NF-κB activation upon ectopic expression. Using coimmunoprecipitation, we identify host proteins interacting with GRA15, including TRAFs, which are key intermediates in the NF-κB pathway that can transduce activating signals through the assembly of signaling complexes, which eventually leads to activation of the NF-κB pathway (26). We also identified particular GRA15 regions and GRA15 TRAF-binding sites that are important for NF-κB activation. Thus, we have identified the mechanism by which GRA15 activates the NF-κB pathway.

## RESULTS

### GRA15 from the type II and type III strains activates NF-κB when expressed ectopically.

**FIG 1:**
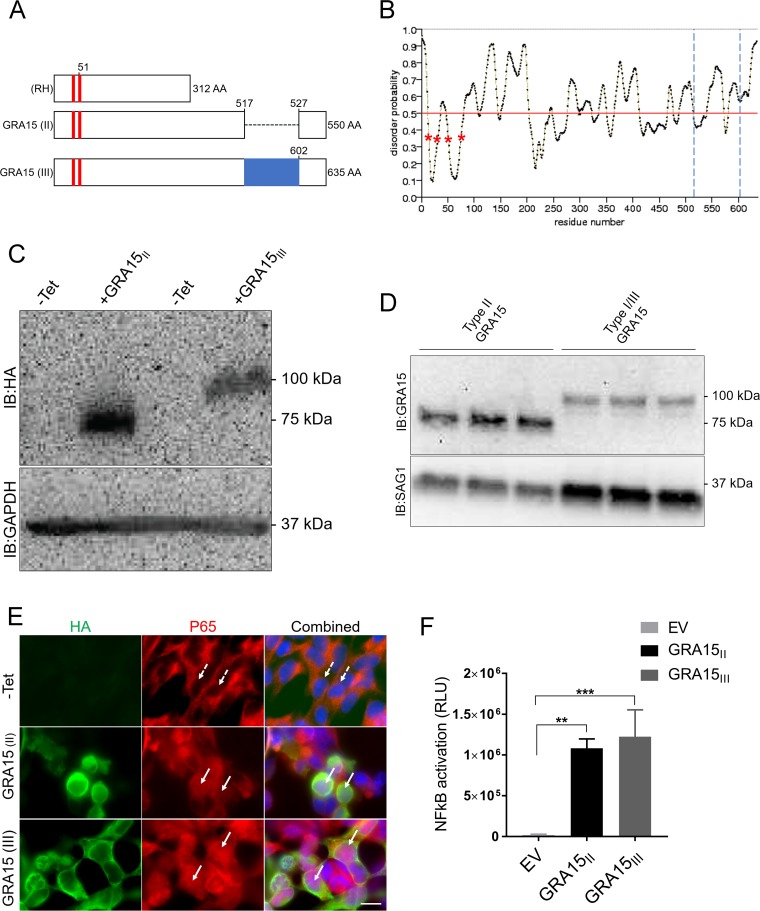
Ectopic expression of either GRA15_II_ or GRA15_III_ is sufficient to activate NF-κB. (A) Schematic showing RH (GRA15), GRA15_II_, and GRA15_III_ protein sequence and alignment. Red boxes indicate two predicted transmembrane domains. The blue box indicates the specific region of GRA15_III_. (B) Structural disorder predictor for GRA15_III_ using the software Protein DisOrder prediction System (PrDOS), with a double asterisk indicating increased structural order across residues 50 to 72. Two blue dashed lines indicate the region specific for GRA15_III_. (C) TREX-293 cells overexpressing GRA15_II_ and RA15_III_ were induced with tetracycline (1** **μg/ml) for 24** **h, fixed with formaldehyde, and stained for p65 (red), HA (green), and Hoechst (blue). The scale bar represents 10** **μm. Arrows indicate nuclei of cells expressing GRA15 and containing nuclear p65. Dashed arrows indicate nuclei of cells containing no nuclear p65. IB, immunoblot. (D) Immunoblot of TREX-293 GRA15_II_- and TREX-293 GRA15_III_-overexpressing cell lysates induced with tetracycline for 24** **h, using an antibody against the HA tag. GAPDH antibody was used as a loading control. (E) Immunoblot on extracellular parasite lysates of type II and type III strains using an antibody against the C terminus of endogenous GRA15, with SAG1 antibody as a loading control. (F) NF-κB reporter HEK293 cells transfected with pcDNA GRA15_II_ and GRA15_III_ or empty vector. The graph shows average luciferase activity from the cell lysate from three independent experiments (*n*** **=** **3), and error bars represent SD. The asterisk indicates significantly higher levels of luciferase activity compared to the empty vector. *P* < 0.0012 for GRA15_II_ and *P* < 0.0006 for GRA15_III_ by one-way ANOVA with Dunnett’s multiple-comparison test.

Type III and type I (GT1 strain) GRA15 protein sequences contain 635 amino acids (aa), while type II GRA15 has 550 aa due to a deletion at the C terminus (Fig. 1A). The type I RH strain has a mutation in the GRA15 gene, leading to a frameshift and early stop codon (14). We observed that even though GRA15 is a relatively disordered protein (27), the amino acids 10 to 29 and 50 to 72 are predicted to be part of two transmembrane regions (Fig. 1B), while no classical signal peptide sequence was predicted (28). Even though no classical signal peptide was predicted, similar to what has been observed for GRA6 (29), we have previously shown that GRA15 is secreted into the PV and localizes to the PVM (14). We, therefore, believe that the first 29 amino acids function as a nonclassical signal peptide. We previously showed that type II strains activate NF-κB more strongly than type III strains (14). We have also shown that other *Toxoplasma*-secreted effectors such as ROP16 and ROP38 can inhibit the NF-κB signaling pathway and that GRA15 expression levels also seem to affect NF-κB activation (19). It is therefore unclear if different GRA15 sequences differ in their capacity to activate NF-κB. We previously showed that GRA15_II_ is sufficient for activating the NF-κB pathway by transfecting HeLa cells with a plasmid capable of expressing a GRA15_II_-GFP (green fluorescent protein) fusion protein (14). However, we were unable to generate stable expression cell lines, likely because the expression of GRA15_II_ was toxic to the cells (not shown). Therefore, to determine if GRA15_II_ and GRA15_III_ can both activate the NF-κB pathway, we engineered stable HEK293-derived (TREX-293) cell lines expressing GRA15_II_ (TGME49_275470, from residues 51 to 550 to exclude the putative signal peptide) or GRA15_III_ (from residues 51 to 631 to exclude the putative signal peptide) with a C-terminal hemagglutinin (HA)-FLAG double-epitope tag under the control of the tetracycline operator. In TREX-293 cells expressing GRA15_II_ or GRA15_III_, expression of these proteins was strictly regulated by tetracycline. GRA15_II_ and GRA15_III_ seemed to have a predominantly cytoplasmic localization (Fig. 1C), although no further attempts were made to determine the exact subcellular localization of these proteins. p65 nuclear translocation indicated that upon induction by tetracycline, both GRA15_II_ and GRA15_III_ were able to activate the NF-κB pathway (Fig. 1C; see Fig. S1A in the supplemental material). We noted that under tetracycline induction, the observed molecular weights of GRA15_II_ and GRA15_III_ (∼75 and ∼90** **kDa, respectively), when ectopically expressed, were higher than the expected predicted sizes (57 and 66** **kDa, respectively [https://www.expasy.org]) (Fig. 1D). We also immunoblotted for the endogenous GRA15 in human foreskin fibroblasts (HFFs) infected with strains expressing either a type II GRA15 (GRA15_II_) or a type I/III GRA15 (GRA15_I/III_) and observed a similar increase in GRA15 size compared to the expected GRA15 size (Fig. 1E). Thus, the larger than the expected size of GRA15 expressed by *Toxoplasma* is not caused by parasite-mediated modification(s) of GRA15. Most likely, it is the particular amino acid composition of GRA15, which is enriched in Pro, Ser, and Thr, that makes it run slower than expected on an SDS-PAGE gel. To determine more quantitatively if there were any differences in the capacity to activate the NF-κB pathway between these two proteins, we transiently transfected HEK293 NF-κB reporter cells (luciferase/GFP) with both GRA15 plasmids. After quantification of the luciferase activity, both proteins activated the NF-κB reporter significantly compared to the empty vector. We did not observe a significant difference between GRA15_II_ and GRA15_III_ in NF-κB activation (Fig. 1F). The amounts of protein in the transient transfections were similar (Fig. S1B). These results indicate that both GRA15_II_ and GRA15_III_ can activate NF-κB.

10.1128/mBio.00808-19.1 FIG S1 (A) TREX-293 cells overexpressing GRA15_II_ and GRA15_III_ were induced with tetracycline (1**** ****μg/ml) for 24 h. The graph represents the mean fluorescence intensity (MFI) of p65 in the nucleus measured in the presence or absence of tetracycline. Shown is a single representative experiment with a number of cells (*n******* *******=******* ******30); error bars represent SD between individual cells. (B) NF-κB reporter HEK293 cells were transfected with pcDNA GRA15_II_ and GRA15_III_ or empty vector and immunoblotted with an antibody against HA (top panel) and GAPDH (bottom panel) for loading control. Download FIG S1, TIF file, 0.7 MB. Copyright © 2019 Sangare et al. 2019 Sangare et al. This content is distributed under the terms of the Creative Commons Attribution 4.0 International license.

### Identification of the GRA15 sequence necessary for NF-κB activation.

**FIG 2:**
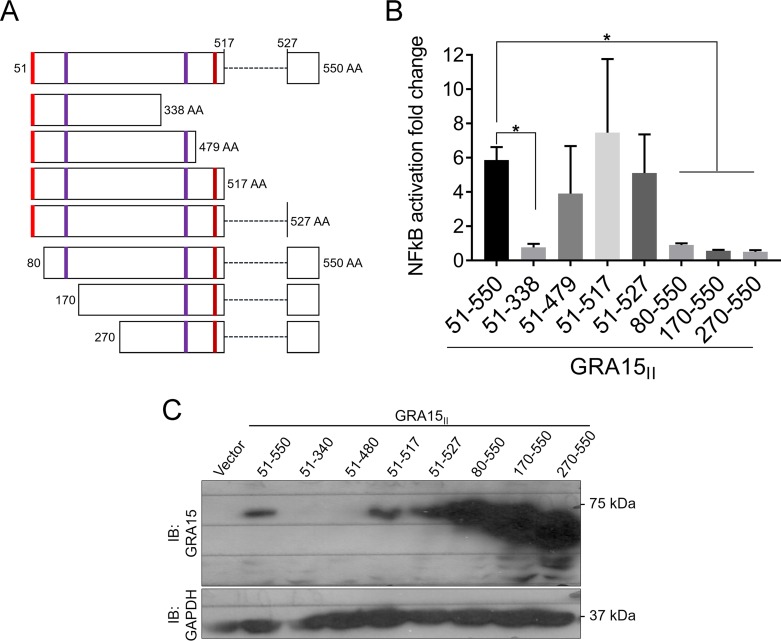
The N-terminal region of GRA15_II_ is required for full NF-κB activation. (A) Schematic showing GRA15_II_ N- or C-terminal truncation mutants. The red box indicates the second predicted transmembrane domain. The purple box indicates the two TRAF2-binding sites, and the brown box indicates the unique TRAF6-binding site. (B) Transient transfections of the NF-κB reporter cell line with GRA15_II_ N- or C-terminal truncation mutants. The graph shows average luciferase fold changes across three independent experiments (*n*** **=** **3), in comparison to empty vector-transfected cells, and error bars represent SD. The asterisk indicates significantly higher levels of luciferase activity compared to cells transfected with GRA15_II-51–550_. *P* < 0.03 for GRA15_51–338_, *P* < 0.04 for GRA15_80–550_, *P* < 0.03 for GRA15_170–550_, and *P* < 0.02 for GRA_270–550_ by one-way ANOVA with Dunnett’s multiple-comparison test. (C) NF-κB reporter cell lines were transiently transfected with GRA15 N- or C-terminal truncation mutants and immunoblotted with an antibody against GRA15 (raised against residues 493 to 510) (top panel) and GAPDH (bottom panel) for the loading control.

BLAST searches with the GRA15 protein sequence did not detect homology to any known proteins or any known protein domains. Therefore, to determine which region of GRA15 is required for GRA15-dependent NF-κB activation, we engineered GRA15 N- and C-terminal truncation mutants. We cloned the full-length GRA15_II-51–550_ without the putative signal peptide (schematic GRA15_II_ [Fig. 2A]), C-terminally truncated GRA15 variants (51–338, 51–479, 51–517, and 51–527), and N-terminally truncated GRA15 variants (80–550, 170–550, and 270–550) fused to GFP in a mammalian expression vector. We transiently transfected both full-length and truncated GRA15 mutants in an HEK293 NF-κB reporter cell line and measured NF-κB activation. Based on the detection of GFP expression, all constructs were successfully expressed and seemed to localize to the host cytoplasm (not shown). The two largest GRA15 C-terminal truncations showed less activation of NF-κB, but this only reached significance for the GRA15_51–338_ construct. The N-terminal truncation mutants had significantly decreased NF-κB activation compared to full-length GRA15_II-51–550_ (Fig. 2B). The N-terminal mutant with the smallest region truncated is GRA15_II-80–550_, indicating that residues 51 to 79 are necessary for GRA15-dependent NF-κB activation. To ensure that the decrease in NF-κB activation was not due to lack of protein expression, we immunoblotted for GRA15 upon transient transfection of HEK293 cells and observed that the N-terminal GRA15 mutants were more strongly expressed than the full-length GRA15_II-51–550_ (Fig. 2C). We were not able to detect the two largest C-terminal GRA15 mutants (GRA15_51–479_ and GRA15_51–338_) on Western blots because the GRA15 antibody was raised against residues 493 to 510. Thus, the decrease in NF-κB activation is specific to the missing sequences in the N-terminal GRA15 sequences and not because of protein expression differences. We observed that the second predicted transmembrane (amino acids 51 to 72) (Fig. 1B) seems to be important for GRA15-mediated activation of NF-κB.

### Identification of GRA15 candidate host-interacting proteins.

**FIG 3:**
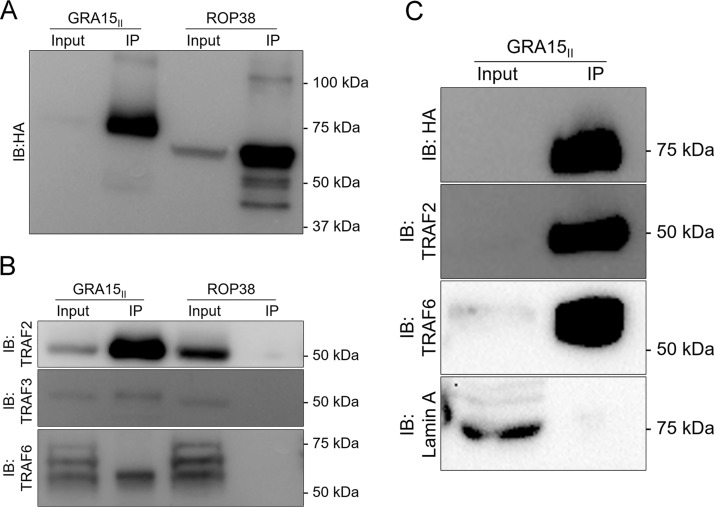
Immunoprecipitation of TREX-293 GRA15_II_ and RH-GRA15_II_. TREX-293 GRA15_II_ and ROP38_I_ were induced with tetracycline (1** **μg/ml), harvested 16** **h postinduction (A, B), or infected for 8** **h with RH parasites expressing HA-tagged GRA15 (C). The cells were lysed for HA immunoprecipitation with magnetic beads. Immunoblotting was performed on 10% input and 30** **μl of the HA magnetic beads with (A) HA antibody, (B) TRAF2, TRAF3, and TRAF6 antibodies, or (C) HA, TRAF2, and TRAF6 antibodies and lamin A as a loading control of the 10% input.

Even though there is evidence that GRA15 requires TRAF6 to achieve full NF-κB activation (14), it is unclear whether GRA15 directly interacts with TRAF6 or acts indirectly through TRAF6 to cause NF-κB activation. Therefore, to determine potential host proteins that interact with GRA15 or are in the same complex with GRA15, we harvested whole-cell lysates from TREX-293 GRA15_II_ 16** **h postinduction with tetracycline. As a negative control, we used TREX-293 expressing ROP38_I_. These lysates were then subjected to immunoprecipitation using the HA antibody, and immunoprecipitates were probed with HA antibody to confirm the presence of each protein in the immunoprecipitates (Fig. 3A). The immunoprecipitates were directly sent for mass spectrometry analysis. To control for potential spurious interactions detected through mass spectrometry, we refined the list of proteins to those identified only in GRA15_II_-overexpressing conditions and excluded proteins detected in immunoprecipitates from ROP38_I_-overexpressing conditions. We identified a list of candidate host proteins that could have direct interactions with GRA15_II_ (Table 1). In the TREX-293 GRA15_II_-expressing cells, we observed that TRAF2, TRAF3, and TRAF6 were detected in GRA15_II_ immunoprecipitates but were absent in ROP38_I_ immunoprecipitates (Table 1). We also observed the baculoviral IAP repeat-containing protein (BIRC2), an E3 ubiquitin-protein ligase know to interact specifically with TRAF2 and for its antiapoptotic function (Table 1). To validate these observations, we immunoblotted the immunoprecipitated GRA15_II_ and ROP38_I_ with antibodies against TRAF2, TRAF3, and TRAF6. We observed a faint band for TRAF3 and a strong signal for TRAF2 and TRAF6 only in the immunoprecipitated GRA15_II_ (Fig. 3B). To confirm these interactions in cells infected with parasites, we immunoprecipitated GRA15 from HEK-293 cells infected with RH parasites expressing HA-tagged GRA15_II_. We immunoblotted the immunoprecipitated GRA15_II_ with antibodies against TRAF2 and TRAF6 and observed a strong signal for these TRAF proteins (Fig. 3C). Thus, GRA15_II_ interacts with host TRAF proteins.

**TABLE 1:** Identification of host interaction partners of GRA15^*a*^

Protein	MW (kDa)	Spectrum counts		Normalized total spectra	
		GRA15	ROP38	GRA15	ROP38
TNF receptor-associated factor 6 (TRAF6)	60	114	0	151	0
TNF receptor-associated factor 2 (TRAF2)	56	106	0	141	0
Baculoviral IAP repeat-containing protein 2 (BIRC2)	70	32	0	44	0
TNF receptor-associated factor 3 (TRAF3)	64	2	0	3	0

a Shown are results from immunoprecipitation (IP) with HA antibody on stable HEK293-derived (TREX-293) cell line expressing GRA15_II_ or ROP38 with a C-terminal HA-FLAG epitope tag under the control of the tetracycline operator. The table represents the putative host interaction partners identified through mass spectrometry analysis specific to GRA15 IP. The spectrum counts indicate the total number of peptide spectra used to identify the protein. The normalized total spectra indicate the normalization of the spectrum counts of protein peptides by the total number of spectra in the sample. MW, molecular weight.

### GRA15 TRAF2-binding sites mediate NF-κB activation.

**FIG 4:**
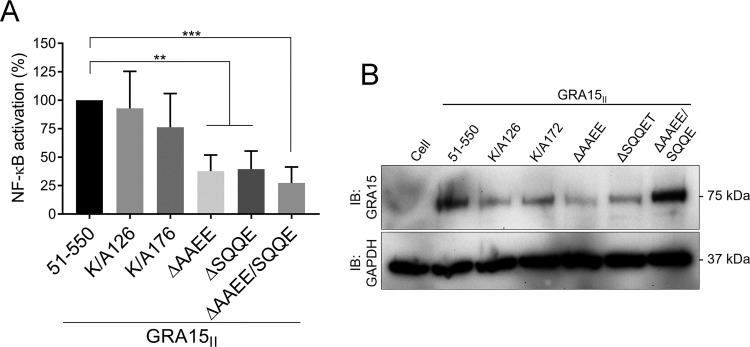
TRAF2-binding sites are required for full NF-κB activation. (A) Transient transfections of the NF-κB reporter cell line with GRA15_II_, mutant for ubiquitinylation sites or TRAF2-binding site mutants. The graph shows the average percentage of luciferase activity of the mutants compared to GRA15_II-51–550_ (indicated as WT) across three independent experiments (*n*** **=** **3), and error bars represent SD. The asterisk indicates significantly higher levels of luciferase activity compared to GRA15_II-51–550_. ns, nonsignificant. *P* < 0.0014 for ΔAAEE, *P* < 0.0010 for ΔSQQE, and *P* < 0.0007 for ΔAAEE/SQQE by one-way ANOVA with Dunnett’s multiple-comparison test. (B) NF-κB reporter cell lines were transiently transfected with the GRA15_II_ mutant for ubiquitinylation sites or TRAF2 binding site mutants and immunoblotted with antibody against GRA15 (raised against residues 493 to 510) (top panel) and GAPDH (bottom panel) for the loading control.

GRA15 does not have homology to any known proteins (www.ncbi.nlm.nih.gov/BLAST). However, our bioinformatic analysis identified two TRAF2-binding sites ([PSAT]x[QE]E), AAEE_160–163_ and SQQE_430–433_, and one TRAF6-binding site (PxExx[FYWHDE]), PGENSY_506–511_, in the GRA15 sequence, which could function as a docking site for TRAF proteins (schematic in Fig. 2A) (30). The mass spectrometry results also identified two ubiquitination sites on the GRA15_II_ sequence: lysine 126 and lysine 172 (see Table S1 in the supplemental material). Therefore, to determine if these putative TRAF2-binding sites and/or the ubiquitination sites are required for GRA15-dependent NF-κB activation, we used Q5 mutagenesis to mutate these sites in the GRA15_II_ full-length sequence. We cloned the full-length GRA15_II-51–550_ without the putative signal peptide (schematic of GRA15_II_ in Fig. 2A), the deletions AAEE_160–163_, SQQE_430–433_, and AAEE_160–163_/SQQE_430–433_, and the substitutions K/A_126_ and K/A_172_ in a mammalian expression vector. We transiently transfected the full-length and GRA15 mutants in the HEK293 NF-κB luciferase reporter cell line to determine differences in NF-κB activation. We did not observe a significant difference in NF-κB activation for the ubiquitination mutants compared to the full-length GRA15_II-51–550_ sequence. However, we observed that the single TRAF2-site mutants had ∼60% decrease in NF-κB activation compared to full-length GRA15 and the double TRAF2-site mutant had an ∼75% decrease in NF-κB activation (Fig. 4A). To ensure that the decrease in NF-κB activation was not due to lack of protein expression, we immunoblotted for GRA15 upon transient transfection of HEK293 cells and observed that the TRAF2-site GRA15 mutants were similarly expressed to the full-length GRA15 (Fig. 4B). Thus, the decrease in NF-κB activation is specific to the missing sequences AAEE_160–163_ and SQQE_430–434_ in GRA15 sequences and is not because of protein expression differences. These data are consistent with GRA15 activating NF-κB through recruitment of TRAF proteins via its TRAF-binding domains.

10.1128/mBio.00808-19.3 TABLE S1 Mass spectrometry results from immunoprecipitation of GRA15_II_ or ROP38_I_. Download Table S1, XLSX file, 0.1 MB. Copyright © 2019 Sangare et al. 2019 Sangare et al. This content is distributed under the terms of the Creative Commons Attribution 4.0 International license.

### TRAF2 is partially needed for GRA15-mediated NF-κB activation.

**FIG 5:**
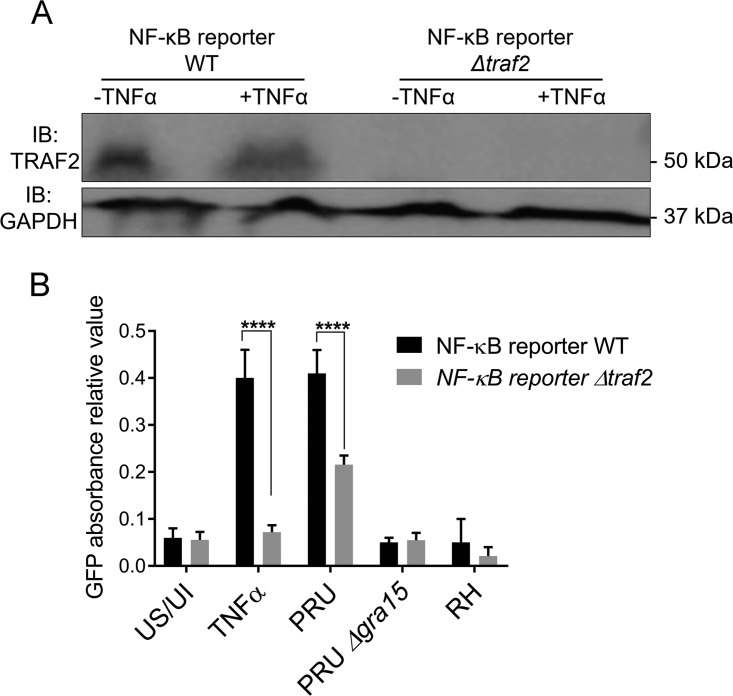
TRAF2 is partially needed for GRA15-mediated NF-κB activation. (A) The Western blot shows the presence of TRAF2 in NF-κB reporter wild-type cells and its absence in NF-κB reporter Δ*traf2* cells stimulated or not with TNF-α. (B) GFP quantitative analysis and comparison between NF-κB reporter wild-type and NF-κB reporter Δ*traf2* cells under different conditions: US/UI (uninfected/unstimulated), TNF-α stimulation only, infection with Toxoplasma gondii strain Pru, Pru Δ*gra15*, and the RH strain. GFP expression is reduced by 47% in the NF-κB reporter Δ*traf2* cells compared to the NF-κB reporter wild type, whereas there is no expression in Pru Δ*gra15* or RH strain-infected cells. The asterisk indicates statistically significant results. TNF-α stimulation of NF-κB reporter wild-type versus NF-κB reporter Δ*traf2* cells: *P* < 0.0001 by two-way ANOVA with Sidak’s multiple-comparison test.

Immunoprecipitation performed on TREX-293 cells overexpressing GRA15_II_ demonstrated that TRAF2 is a binding partner of GRA15. To elucidate the cross talk between GRA15 and TRAF2, we performed additional experiments, utilizing an HEK293 NF-κB–green fluorescent protein (GFP) reporter cell line. We used CRISPR/Cas9 to generate an indel in TRAF2 (see Fig. S2 in the supplemental material) in this reporter cell line and isolated a clonal line that no longer expressed TRAF2 (Fig. 5A). Both the wild-type and the TRAF2 knockout reporter lines (Δ*traf2*) were infected with a type II strain (Pru), a type II Δ*gra15* strain, and the type I RH strain, which does not express GRA15. (Note that none of these strains expresses GFP.) To measure the activation of the NF-κB pathway, we performed a quantitative analysis of GFP expression. GFP levels were undetectable in noninfected and nonstimulated cells. There was a significant (∼47%) decrease in expression of GFP between the Δ*traf2* and the wild-type cells when they were infected with type II (Fig. 5B). Infection with the type II Δ*gra15* or the RH strain did not lead to any GFP expression. As shown by others (31), Δ*traf2* cells were susceptible to TNF-α, and all cells died upon addition of TNF-α (not shown), and therefore TNF-α did not induce NF-κB activation in these cells (Fig. 5B). These results show that the activation of the NF-κB pathway by GRA15 is partially dependent on TRAF2.

10.1128/mBio.00808-19.2 FIG S2 (A) Sequencing data from NF-κB reporter Δ*traf2* and NF-κB reporter wild-type genomic DNA compared to the reference sequence of the *TRAF2* gene. The red arrow indicates the insertion of a nucleotide at position 114 of the first exon of the *TRAF2* gene. (B) Microscopy shows a decrease in the GFP expression between the NF-κB reporter wild-type (top panel) and NF-κB reporter Δ*traf2* cell lines (bottom panel) after infection with the *Toxoplasma* Pru strain. Download FIG S2, TIF file, 2.3 MB. Copyright © 2019 Sangare et al. 2019 Sangare et al. This content is distributed under the terms of the Creative Commons Attribution 4.0 International license.

## DISCUSSION

We observed that upon ectopic expression of either type II or type III GRA15, there was significant NF-κB activation compared to noninduced controls. In contrast, upon infection of human or murine cells, type II strains strongly activate NF-κB, while type III strains induce very weak or no NF-κB activation (14). Thus, it is likely that the low expression level of GRA15 in type III strains and the presence of type III strain effectors that have an inhibitory effect, such as ROP38, which is highly expressed in type III strains and inhibits NF-κB and ROP16, which can also inhibit NF-κB (19), explain the absence of NF-κB activation by type III strains.

We identified several host proteins that may directly interact with GRA15 or are present in a complex with GRA15. The most promising interaction partners were TRAFs, which coimmunoprecipitated with GRA15_II_ ectopically expressed in TREX cells but were absent in all other immunoprecipitations. TRAF2 and TRAF6 are both involved in the canonical NF-κB activation pathway (32), whereas TRAF2 and TRAF3 are involved in nonredundant negative regulatory roles in the alternative NF-κB activation pathway (33, 34). Upon exposure to classical activating stimuli such as TNF-α, TRAF2 becomes polyubiquitinated and activates a downstream kinase, RIPK, to cause activation of the IKK complex (35). Other canonical activating stimuli, such as lipopolysaccharide (LPS), cause oligomerization of TRAF6 and activation of its E3 ligase activity, which leads to recruitment and activation of TAK1, a downstream kinase. TAK1 then binds to the regulatory subunit of the IKK complex to activate IKKα/β, causing phosphorylation of IκBα and allowing nuclear translocation of p65/p50 heterodimers to occur. We observed a faint TRAF3 band in the GRA15 immunoprecipitate in TREX cells, suggesting that TRAF3 is not a direct interactor of GRA15. TRAF3 was demonstrated to physically interact with the NF-κB-inducing kinase (NIK) and mediate its ubiquitination and degradation (36). Thus, noncanonical NF-κB activation is associated with TRAF3 degradation and concomitant accumulation of NIK (36). Another study demonstrated that NIK degradation is cross-regulated by a complex consisting of TRAF3/TRAF2/cIAP1/cIAP2 (34). The GRA15 primary sequence contains two TRAF2 binding motifs (AAEE_160–163_ and SQQE_430–433_) and one TRAF6 binding motif (PGENSY_506–511_), and we show that the TRAF2 motifs play a role in GRA15-mediated NF-κB activation. Likely, the remaining activation of NF-κB we observed in the double TRAF2 GRA15 mutant is due to the remaining TRAF6 binding site. Taken together, GRA15 TRAF-binding sites are functional and likely complementary by recruiting both TRAF2 and TRAF6.

GRA15-mediated NF-κB activation seems remarkably similar to what has been described as the mechanism of NF-κB activation by the Epstein-Barr virus (EBV), which can cause Burkitt lymphoma. EBV encodes the effector LMP1 (latent membrane protein 1), which persistently activates NF-κB by mimicking CD40 signaling (37). The CD40 cytoplasmic domain has one TRAF6- and two TRAF2-binding sites, which are important for its function (38). LMP1 is a six-membrane-spanning molecule that contains two C-terminal activation (CTAR) domains, CTAR1 and CTAR2, which activate the alternative and canonical NF-κB pathways, respectively. CTAR1 interacts with TRAF2 and TRAF3 to activate the alternative NF-κB pathway, and this alternative activation is dependent on the TRAF-binding site in CTAR1 (39). On the other hand, CTAR2 interacts with TRAF6, leading to activation of the canonical NF-κB pathway, dependent on eight residues within CTAR2 (40). Constitutive activation of the NF-κB pathway in B-cells, which are the preferred cell type infected by EBV, leads to the malignant transformation of these cells (41). Although we previously have seen no evidence of GRA15 activating the alternative NF-κB pathway in human foreskin fibroblasts (14). It is possible that such activation could take place in different cell types. Interestingly, it has been shown that activation of CD40 signaling in both hematopoietic and nonhematopoietic cells infected with *Toxoplasma* leads to autophagy-mediated destruction of the parasitophorous vacuole (42). It will be of interest to determine if GRA15 signaling can also affect the autophagy pathway.

## MATERIALS AND METHODS

### Plasmids.

The vector pcDNA-LIC-HF was a gift from M. A. Hakimi and A. Bougdour. Primers were designed to amplify after the predicted signal peptide to the predicted stop codon. Forward primers to amplify GRA15 (5′-T*GGCTGGTGCTGGTGCCCATATAATTCGGTGGCTTGGGTATCTT-3′) together with reverse primers (5′**-GCTCCGGCTCCTGCCCCAGC**TGGAGTTACCGCTGATTGTGTG-3′) contained ligation-independent cloning (LIC) sequences (in boldface) and were used to amplify GRA15 from PRU (II) and CEP (III) genomic DNA. Forward primers to amplify ROP38_I_ (5′-**TGGCTGGTGCTGGTGCCCAT**CATGGCAGCAGCACTGATGGATCAG-3′) together with reverse primers (5′-**GCTCCGGCTCCTGCCCCAGC**AAATTGATGCGTTCTTATCCGA-3′) contained LIC sequences (in boldface) and were used to amplify ROP38 from RH genomic DNA. PCR products were treated with T4 DNA polymerase (using only TTP at 100** **mM). The pcDNA-LIC-HF vector was digested with SmaI and treated with T4 DNA polymerase (using only ATP at 100** **mM) to generate long overhangs. The PCR fragment and vector were then annealed for 15 min at room temperature, generating expression vectors with *Toxoplasma* genes C-terminally tagged with HA-FLAG.

The vector pIC242 was a gift from I. Cheeseman (Whitehead Institute, Cambridge, MA). The GRA15_II_ full-length protein (51 to 550 aa) was amplified and inserted into pIC242 by restriction/ligation, expressing GRA15_II_ mutants as N-terminal fusion GFP proteins. Expression of GFP fusion proteins was promoted by the endogenous retroviral long terminal repeats. GRA15 truncation and mutation constructs were amplified from the pIC242 GRA15_II_ full-length protein (51 to 550 aa) using specific primers (Table 2) and confirmed by sequencing.

**TABLE 2:** GRA15 primers used in this study^*a*^

GRA15 truncation or mutation	5′→3′ primer	
	Forward	Reverse
GRA15_51–550_	*CTC GAG* **ATAATTCGGTGGCTTGGGTATCTT**	*GAA TT*C **TGGAGTTACCGCTGATTGTGTG**
GRA15_51–338_		*GAA TT*C TCA ATT TGA GTA CGT TAA CCT CGC CTC CGT CTC
GRA15_51–479_		*GAA TT*C TCA ATG CGG AGA CGA GAT GGG TTG TGT ACG TGA
GRA15_51–517_		*GAA TT*C TCA CTC AGT TGG TAC AGA GTA GTA AGA GTT TTC
GRA15_51–527_		*GAA TT*C TCA CCT TTG GGG ACC TGG ATC AGA GAT CGT CCG
GRA15_80–550_	*CTC GAG* TCC GAC TCA GTG CGG GAA ACA CGG GAA ACA CGG CGA GGC	
GRA15_170–550_	*CTC GAG* GAC AGA AAA GTT CCG GAG GGT GCC CAA CTC	
GRA15_270–550_	*CTC GAG* CCA ACG GCA CCA GCA CCA GCG ACC GCC ACT AGC AAC	
K/A1126	CGTCAACCGATGGAGCGTCTGAGAGTGAAC	TGTCAGCGCCGCAGCCGCCGT
K/A172	GTCCGAGAGACAGAGCAGTTCCGGAGGGTG	TCGAAGAGCGTTCTTCCGCCGCCTTGC
ΔAAEE	CGCTCTTCGAGTCCGAGAGACAGAAAAG	CTTGCGCGTCGCGAATGGGGAA
ΔSQQE	CTCCCTGTGGTAGAAAATGCGACTTTC	TAGTGGAAGTTGGCTTTGCACGGC

a 5′ XhoI and 3′ EcoRI are in italic. The forward primer sequence common for all the C-terminal truncations and the reverse primer sequence common for all the N-terminal truncations are in boldface.

### Inducible TREX-293 cell line construction.

The TREX-293 cell line was a gift from J. Niles (MIT, Cambridge, MA). TREX-293 cells were seeded at 75% confluence and cotransfected with expression vector pcDNA-LIC-GRA15_II_-HF or ROP38_I_-HF and a puromycin resistance vector (ratio of 10:1), using the X-tremeGENE 9 DNA transfection reagent (Roche). Cells were split 2** **days posttransfection and subjected to puromycin (Calbiochem) selection at 1** **μg/ml. Foci were picked and expanded at least 1 week postselection, and positive foci were selected through HA expression using immunofluorescence and immunoblotting.

### Immunofluorescence.

TREX-293 cells overexpressing GRA15_II_ or GRA15_III_ were plated at 80% confluence in 24-well coverslip plates, induced with tetracycline (Sigma-Aldrich) at 1** **μg/ml for 24** **h, fixed with formaldehyde for 20 min, and blocked with blocked with phosphate-buffered saline (PBS) with 3% (wt/vol) bovine serum albumin (BSA) and 5% (vol/vol) goat serum. Coverslips were incubated with anti-HA (1:500 [Roche]) and anti-p65 (1:500 [Santa Cruz SC-109]) at 4°C overnight, and fluorescent secondary antibody and Hoechst stain were used for HA and p65 and DNA visualization, respectively.

### Transient transfections.

NF-κB reporter HEK293 cells (luciferase/GFP) (System Biosciences) were seeded at 7.5** **×** **10^5^ cells per well in a 6-well plate and incubated for 4** **h at 37°C. Cells were subsequently transiently transfected with XtremeGene9 transfection reagent (Roche) with pcDNA-LIC-GRA15_II_-HF, GRA15_III_-HF, or pIC242-GRA15_II_ (full-length and mutants) and incubated for 24** **h at 37°C. Cells were lysed in 100** **μl Promega Passive lysis buffer, and luciferase activity was measured in lysates according to the manufacturer’s instructions. Data from conditions with comparable transfection efficiencies were used.

### Coimmunoprecipitation.

TREX-293 cells overexpressing GRA15_II_ or ROP38_I_ were grown in a T175 flask until 100% confluence and induced with tetracycline (1** **μg/ml) for 24** **h. HEK-293 wild-type cells were grown in a T175 flask until 100% confluence and infected for 8** **h with RH expressing GRA15_II_. Cells were then scraped in ice-cold PBS. The cells were centrifuged and resuspended them in 1** **ml of lysis buffer (HEPES, 10** **mM [pH 7.9]; MgCl_2_, 1.5 mM; KCl, 10** **mM; EDTA, 0.1 mM; dithiothreitol [DTT], 0.5 mM; NP-40, 0.65%; protease inhibitor cocktail [Roche]; phenylmethylsulfonyl fluoride [PMSF], 0.5 mM) for 30 min at 4°C. The lysate was centrifuged for 30 min at 18,000** **×** ***g* at 4°C. The samples were incubated with 100 or 30** **μl of magnetic beads coupled with HA antibodies (Thermo Scientific) overnight at 4°C, rotating. The beads were washed 3 times with Tris-HCl (10** **mM [pH 7.5]), NaCl (150** **mM), Triton X-100 (0.2%), PMSF (0.5 mM), and a protease inhibitor cocktail (Roche) and then one more time with Tris-HCl (62.5 mM [pH 6.8]), and the beads were resuspended in 100 or 40** **μl of this buffer.

### Western blotting.

Ten percent input lysate and 20** **μl of the magnetic beads coupled with antibodies to HA of each sample were used to run SDS-PAGE. The proteins were transferred to a polyvinylidene difluoride (PVDF) membrane, blocked for 30 min with TBST (Tris-buffered saline with Tween 20)–5% nonfat dry milk. The membrane was blotted overnight at 4°C with rat antibody against HA (3F10, 1:500 dilution [Roche]) and TRAF2, TRAF3, TRAF6, and lamin A rabbit antibodies (sc-876, sc-1828, sc-7221, and sc-293162, respectively, 1:200 [Santa Cruz]), followed by respective secondary horseradish peroxidase (HRP)-conjugated antibodies. NF-κB reporter HEK293 cells transfected with GRA15 constructs were lysed with lysis buffer, boiled for 5 min, and subjected SDS-PAGE. Proteins were transferred to a PVDF membrane, blocked for 30 min with TBST plus 5% nonfat dry milk, incubated with affinity-purified rabbit polyclonal antibodies to GRA15 (raised against the peptide with the amino acid sequence of GRA15_493–510_, 1** **μg/ml [YenZym Antibody, San Francisco, CA]) (19) or antibodies against mouse GAPDH (sc-32233, 1:500 [Santa Cruz]), overnight at 4°C, followed by the respective secondary HRP antibodies. TREX-293 GRA15_II_ or GRA15_III_ was induced or not with tetracycline (1** **μg/ml) for 24** **h. The cells were lysed and subjected to SDS-PAGE. After transfer, the PVDF membrane was incubated with rat antibody against HA (Roche) and mouse GAPDH antibodies (Santa Cruz) for 1** **h at room temperature, followed by the respective secondary HRP-conjugated antibodies. The parasite lysates were lysed and subjected to SDS-PAGE. After transfer, the PVDF membrane was incubating with rabbit antibody against SAG1 for 1** **h at room temperature, followed by the respective secondary HRP-conjugated antibody.

### Mass spectrometry-based proteomics.

The magnetic beads coupled with antibodies against HA were sent to the Proteomic Core Facility of the University of California Davis for mass spectrometry analysis. Briefly, the proteins were digested using Promega modified trypsin overnight at room temperature on a gently shaking device. The resulting peptides were analyzed by online liquid chromatography-tandem mass spectrometry (LC-MS/MS) with Q-Exactive. All MS/MS samples were analyzed using X! Tandem [The GPM, thegpm.org; version X! Tandem Alanine (2017.2.1.4)]. X! Tandem was set up to search the uniprotHSTG_crap database, assuming the digestion enzyme trypsin. X! Tandem was searched with a fragment ion mass tolerance of 20 ppm and a parent ion tolerance of 20 ppm. Glu→pyro-Glu of the N terminus, ammonia loss of the N terminus, Gln→pyro-Glu of the N terminus, deamidation of asparagine and glutamine, oxidation of methionine and tryptophan, dioxidation of methionine and tryptophan, and dicarbamidomethyl of lysine were specified in X! Tandem as variable modifications. Scaffold (version Scaffold_4.8.6; Proteome Software, Inc., Portland, OR) was used to validate MS/MS-based peptide and protein identifications. Peptide identifications were accepted if they could be established at greater than 50.0% probability by the Scaffold local false-discovery rate (FDR) algorithm. Peptide identifications were also required to exceed specific database search engine thresholds, and X! Tandem identifications were also required at least. Protein identifications were accepted if they could be established at greater than 9.0% probability to achieve an FDR less than 5.0% and contained at least 1 identified peptide. Protein probabilities were assigned by the Protein Prophet algorithm (43). Proteins that contained similar peptides and could not be differentiated based on MS/MS analysis alone were grouped to satisfy the principles of parsimony. Proteins sharing significant peptide evidence were grouped into clusters.

### Construction of a stable NF-κB reporter Δ*traf2* HEK293 cell line.

A TRAF2 knockout cell line was made in NF-κB GFP reporter HEK293 cells (System Biosciences) using CRISPR/Cas9. Wild-type NF-κB reporter HEK293 cells are expressing green fluorescent protein (GFP) when the NF-κB pathway is activated. The vector pSpCas9 (BB)-2A-Puro (PX459) was purchased from Addgene (plasmid 48139). Guide RNAs (gRNAs) were designed and selected based on MIT’s site CRISPR Design Tool (http://crispr.mit.edu/). The gRNAs (20 nucleotides in length) were designed to target the first exon of the TRAF2 gene. The forward gRNA for TRAF2 (5′-CACCGCCTGCAGAAACGTCCTCCGC-3′) and the reverse guide RNA (5′-AAACGCGGAGGACGTTTCTGCAGGC-3′) were selected as the guides with the lowest score of “off-target” events. After the appropriate guides were designed, they were cloned into the pSpCas9 (BB) vector for coexpression with Cas9. All of the procedures were based on the protocol by Ran et al. (44). NF-κB reporter cells were plated at 7 × 10^4^ cells per well in a 24-well plate and incubated for 24 h at 37°C. They were then transiently transfected with the guide RNAs/CRISPR/Cas9 plasmid using the X-tremeGENE 9 protocol as per the manufacturer’s instructions (Roche). At 48 h posttransfection, cells were plated in a new 24-well plate, and selection with 1 μg/ml puromycin was initiated. Cells were left under puromycin selection for 3 days. On the third day of selection, isolation of the clonal population was initiated using serial dilution. The clonal population was isolated after 12 days. Sequence analysis of the genomic DNA detected the insertion of a nucleotide at position 114 of the first exon of TRAF2, and Western blot analysis further verified the TRAF2 knockout (Fig. S2).

### Stable NF-κB reporter Δ*traf2* cell line infection with *Toxoplasma* parasites.

NF-κB reporter wild-type and NF-κB reporter Δ*traf2* HEK293 cells were seeded at 80% confluence in a 96-well plate and incubated for 4** **h at 37°C. Subsequently, the cells were infected with the Pru, Pru Δ*gra15*, or RH strain (multiplicity of infection [MOI] of 2), stimulated with TNF-α (20** **ng/ml), or left uninfected/unstimulated. The GFP absorbance of each 96-well plate was measured.

### Statistical analysis.

All statistical analyses were performed using Graph Pad Prism version 7.0. All the data presented are mean ± standard deviation (SD), and the exact *n* values are mentioned in each of the figure legends. For all calculations, *P* values of <0.05 are considered significant. For a one-variable test with two groups, the two-way analysis of variance (ANOVA) was used, followed with Sidak's multiple-comparison test. For more than three groups with one variable, one-way ANOVA was followed by Dunnett's multiple-comparison test.
