# Supplementary figures and images for: Toxoplasma GRA15 Activates the NF-κB Pathway through Interactions with TNF Receptor-Associated Factors
Source: mBio. 2019 Jul 16;10(4):e00808-19. doi: 10.1128/mBio.00808-19 (PMC6635525; doi:10.1128/mBio.00808-19)

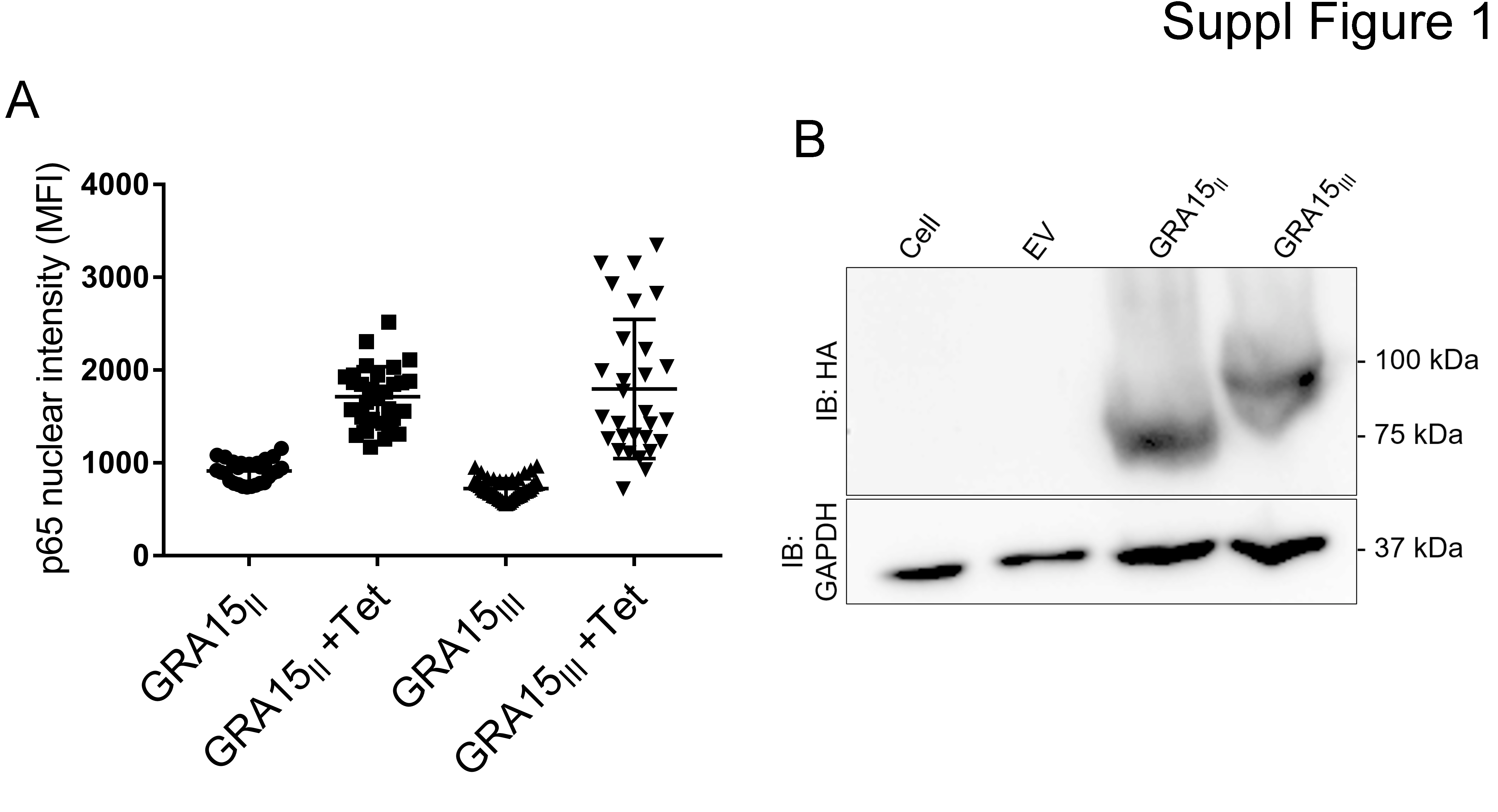

Supplement: FIG S1 [file mBio.00808-19-sf001.tif]

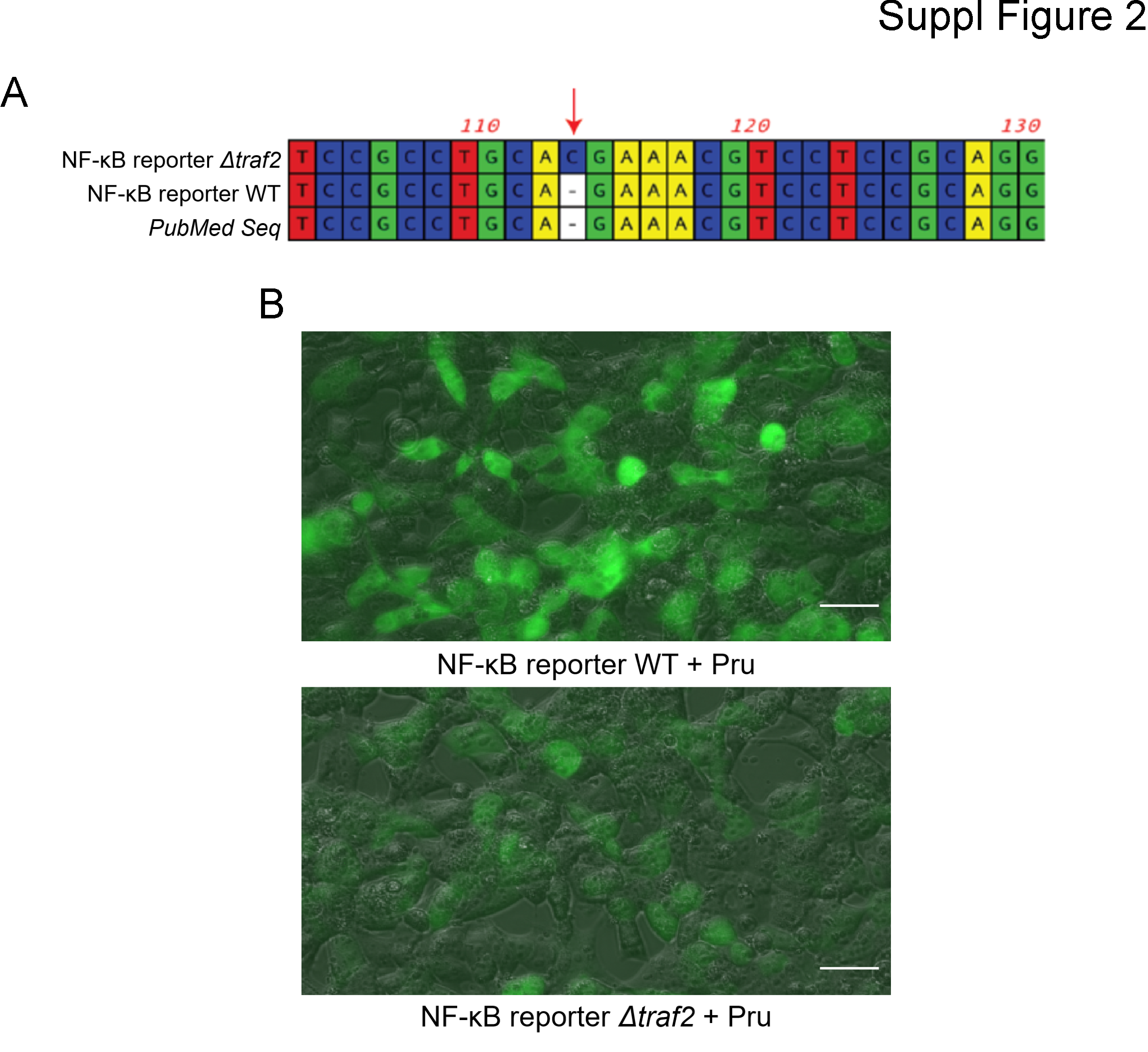

Supplement: FIG S2 [file mBio.00808-19-sf002.tif]
